# Evaluation of alcohol-free mouthwash for studies of the oral microbiome

**DOI:** 10.1371/journal.pone.0284956

**Published:** 2023-04-27

**Authors:** Yukiko Yano, Emily Vogtmann, Alaina H. Shreves, Stephanie J. Weinstein, Amanda Black, Norma Diaz-Mayoral, Yunhu Wan, Weiyin Zhou, Xing Hua, Casey L. Dagnall, Amy Hutchinson, Kristine Jones, Belynda D. Hicks, Kathleen Wyatt, Michelle Brotzman, Nicole Gerlanc, Wen-Yi Huang, Paul S. Albert, Nicolas Wentzensen, Christian C. Abnet

**Affiliations:** 1 Division of Cancer Epidemiology & Genetics, National Cancer Institute, Bethesda, Maryland, United States of America; 2 Department of Epidemiology, Harvard T.H. Chan School of Public Health, Boston, Massachusetts, United States of America; 3 Leidos Biomedical Research, Inc., Frederick National Laboratory for Cancer Research, Frederick, Maryland, United States of America; 4 Public Health Sciences Division, Fred Hutchinson Cancer Research Center, Seattle Washington, United States of America; UAE University: United Arab Emirates University, UNITED ARAB EMIRATES

## Abstract

Oral bacteria play important roles in human health and disease. Oral samples collected using ethanol-containing mouthwash are widely used for oral microbiome studies. However, ethanol is flammable and not ideal for transportation/storage in large quantities, and some individuals may avoid ethanol due to the burning sensation or due to various personal, medical, religious, and/or cultural factors. Here, we compared ethanol-free and ethanol-containing mouthwashes using multiple microbiome metrics and assessed the stability of the mouthwash samples stored up to 10 days before processing. Forty volunteers provided oral wash samples collected using ethanol-free and ethanol-containing mouthwashes. From each sample, one aliquot was immediately frozen, one was stored at 4°C for 5 days and frozen, while the third aliquot was stored for 5 days at 4°C and 5 days at ambient temperature to mimic shipping delays and then frozen. DNA was extracted, the 16S rRNA gene V4 region was amplified and sequenced, and bioinformatic processing was performed using QIIME 2. Microbiome metrics measured in the two mouthwash types were very similar, with intraclass correlation coefficients (ICCs) for alpha and beta diversity metrics greater than 0.85. Relative abundances of some taxa were significantly different, but ICCs of the top four most abundant phyla and genera were high (> 0.75) for the comparability of the mouthwashes. Stability during delayed processing was also high for both mouthwashes based on alpha and beta diversity measures and relative abundances of the top four phyla and genera (ICCs ≥ 0.90). These results demonstrate ethanol-free mouthwash performs similarly to ethanol-containing mouthwash for microbial analyses, and both mouthwashes are stable for at least 10 days without freezing prior to laboratory processing. Ethanol-free mouthwash is suitable for collecting and shipping oral wash samples, and these results have important implications for planning future epidemiologic studies of the oral microbiome.

## Introduction

The human oral cavity is estimated to harbor 700 or more bacterial species, in addition to fungi, viruses, archaea, and protozoa, making the oral microbiome one of the most complex microbial communities found in the human body [1, 2]. The number of oral microbiome studies have increased rapidly in the last decade, reflecting the growing interest in studying the potential implications of the oral microbiome on human health and disease [3]. Oral bacteria are involved in the development of dental caries and periodontal disease, the two most common oral diseases [4, 5], and oral microbes are also suspected to play a role in the etiology of various systemic diseases, such as diabetes, cardiovascular disease, rheumatoid arthritis, Alzheimer’s disease, and certain cancers [3, 6–8].

Different types of samples have been used to study the oral microbiome, including saliva, supra- and sub-gingival plaque, and oral swabs from the cheeks, gums, and tongue [9,10]. Among them, saliva and oral wash samples are particularly attractive for large epidemiologic investigations since they are noninvasive and relatively inexpensive to collect, and this method is often employed to collect human genomic DNA for genotyping [11]. Commercial kits have been used for collecting and stabilizing saliva in previous oral microbiome studies [12, 13], but these commercial kits can be costly for large-scale collections. Oral wash samples can be collected at a lower cost using commercially available mouthwashes, such as Crest® Scope® (Scope®), which has been demonstrated to be suitable for oral microbiome analysis [12, 14, 15]. Scope® mouthwash collections have been shown to be stable at room temperature for at least 4 days [12], so oral washes can be self-sampled at home and transferred without freezing [11, 16], which facilitates large-scale collections. There are also existing resources from prospective cohort studies that have archived Scope® mouthwash samples stored in biorepositories (oftentimes originally collected for human genomic analysis), which have been used to conduct nested case-control studies examining the relationship between the oral microbiome and various disease outcomes [17–20].

Although standard Scope® mouthwash is widely used to collect oral washes in epidemiologic studies, there are some practical considerations for such ethanol-containing mouthwashes in future collections. The ethanol in these mouthwashes serves as a solvent and preservative [21]. However, exposure to ethanol may be undesirable for certain populations, such as people who are sensitive to the burning sensation, people with certain religious beliefs or cultural norms, and people with contraindications such as current/recovering alcoholics, patients with oral mucosal injuries, and immunocompromised individuals [21–23]. Another important consideration is that ethanol is a flammable liquid, and so it may not be ideal to transport or store large quantities of ethanol-containing mouthwash.

Alcohol-free (i.e., ethanol-free) mouthwashes do not contain ethanol and provide an alternative method for oral wash collections. Aside from ethanol, alcohol-free formulations of mouthwashes contain many of the same ingredients as ethanol-containing mouthwashes, generally consisting of water, humectant (e.g., glycerin), surfactant (foaming agent), flavor, sweeteners, color, preservatives, and active ingredients with antimicrobial properties for plaque and gingivitis control (e.g., chlorhexidine gluconate, cetylpyridinium chloride, essential oils) [24–26]. However, ethanol-free mouthwashes have not been used as widely in epidemiologic studies, and it is unclear whether ethanol-free mouthwashes are suitable for oral microbiome investigations. One previous study has compared the oral microbiome measured in an ethanol-containing mouthwash and an ethanol-free mouthwash with the OMNIgene ORAL kit as a reference [14]. This study indirectly showed that the two mouthwashes performed more similarly to each other than to the OMNIgene ORAL kit based on alpha and beta diversity and relative abundances of taxa. However, no direct comparisons have been made between ethanol-containing and ethanol-free mouthwashes for oral microbiome studies, and the stability of ethanol-free mouthwash during extended periods without freezing has not been evaluated.

Here, we tested the use of an ethanol-free mouthwash to collect oral washes for studying the oral microbiome. We evaluated the comparability of ethanol-free and ethanol-containing mouthwashes based on multiple microbiome metrics. We also assessed the sensitivity of mouthwash samples to processing delays by storing samples for up to 10 days to mimic shipping delays that may occur during collections shipped via mail or other carriers. The results of this study will be informative for planning oral wash collections for future microbiome studies.

## Materials and methods

### Study population

Forty healthy volunteers were recruited from the Research Donor Program at the Frederick National Laboratory for Cancer Research (a federal facility in Frederick, MD) under an approved protocol (ClinicalTrials.gov NCT00339911). Eligible individuals were employees of the Frederick National Laboratory or Fort Detrick community, between 40–65 years old, weighed 110 pounds or more, did not have dentures or dental implants, and were in excellent health without known heart, lung, kidney, bleeding disorders, infectious disease or other chronic illnesses. Written informed consent was obtained from all participants. Study participants completed questionnaires providing information about demographics, oral hygiene practices, overall oral health, and recent antibiotic use. An equal number of men (n = 20) and women (n = 20) were recruited. The mean age of the participants was 52.4 years (standard deviation [SD] 7.21), and most participants were white (87.5%). The majority of participants self-reported good to excellent oral health (90.0%) with no gum disease (80.0%). Approximately half of the participants had no lost teeth (55%), and most subjects had had dental caries (92.5%). All participants reported practicing daily toothbrushing. Approximately half (52.5%) of the participants reported using a mouthwash product (i.e., ethanol-containing, ethanol-free, chlorhexidine, fluoride, peroxide, cetylpyridinium chloride, sensitive teeth, or dry mouth mouthwash) at least once a week within the month prior to sample collection. One subject reported using antibiotics within 1–4 weeks prior to sample collection, and five subjects received professional dental cleaning within the month prior to sample collection (12.5%). There were no missing numbers in the questionnaire data mentioned above.

This study is reported according to the Strengthening The Organization and Reporting of Microbiome Studies (STORMS) guidelines for human microbiome research (completed checklist available in S2 File) [27].

### Oral sample collection

Each study participant provided two self-sampled oral wash specimens: one with ethanol-free mouthwash (Crest® Pro-Health™, Clean Mint, Alcohol Free, Procter & Gamble, Cincinnati, OH) and another with ethanol-containing mouthwash (Crest Scope®, Procter & Gamble, Cincinnati, OH). The ingredients of the ethanol-free mouthwash contained cetylpyridinium chloride (0.07%), water, glycerin, flavor, poloxamer 407, sodium saccharin, methylparaben, sucralose, propylparaben, and Blue 1. For the ethanol-containing mouthwash, the ingredients were water, ethanol (15 wt%), glycerin, flavor, polysorbate 80, sodium saccharin, sodium benzoate, cetylpyridinium chloride, benzoic acid, Blue 1, and Yellow 5. Participants were provided two home-collection kits containing the mouthwashes along with instructions for collecting the samples. The two mouthwash samples were collected on two consecutive days, where half of the participants were randomly assigned to provide ethanol-free mouthwash samples on the first day and ethanol-containing mouthwash samples on the second day, and vice versa for the other half of the participants. Participants were instructed to pour 10 mL of the mouthwash into a sterile screw-cap container (with a fill line), which they then poured into their mouth and swished for 30 seconds. After this, participants spit the mouthwash back into the screw-cap container. Mouthwash samples were collected in the morning and kept cold (~4°C) with an ice pack before being transferred to the laboratory on the same day for aliquoting and storage. Participants were instructed not to drink, eat, chew gum, use tobacco products, or brush their teeth at least one hour prior to oral wash collection.

Upon receiving the mouthwash samples at the laboratory, three equal-volume aliquots (3 mL) were created from each sample. One aliquot was immediately processed and frozen, and two aliquots were stored for 5 and 10 days prior to processing and freezing. Stored samples were kept at 4°C through 5 days, after which the “10 day” samples were moved to ambient temperature (22°C) for the remaining 5 days. For processing, all samples were centrifuged (3,000 x g for 10 min at 4°C) to obtain the cell pellet, which was resuspended in 2.5 mL of 1X Tris-EDTA (pH 8.0) and stored as two 1 mL aliquots at -80°C until analysis.

### DNA extraction, amplification, and sequencing

Samples were processed in three batches of 96 samples, with samples from the same individual (3 freezing time-points x 2 mouthwashes = 6 samples per subject) kept in the same batch. Each extraction batch contained the following quality control samples: one chemostat community sample [28], and six extraction and sequencing blanks. DNA extraction was performed with the MagMAX DNA Multi-Sample Ultra 2.0 Kit (Thermo Fisher Scientific, Waltham, MA) on the KingFisher Flex Purification System (Thermo Fisher Scientific, Waltham, MA). Proteinase K-enhancer solution (40 *μ*L) was added to sample aliquots (400 *μ*L of resuspended cell pellet), mixed for 1 min, and Proteinase K (40 *μ*L) was added and mixed for 20 min at 70°C to digest proteins and other contaminants. Then, a lysis/binding buffer (400 *μ*L) and magnetic binding beads (40 *μ*L) were added and mixed for 5 min to simultaneously lyse cells and capture DNA on the bead surfaces. Bound DNA was collected and washed in a wash buffer (1,000 *μ*L) for 2 min, 80% ethanol (1,000 *μ*L) for 80 sec, again with 80% ethanol (500 *μ*L) for 30 sec, and SPM buffer (500 *μ*L) for 1 min. The beads were dried for 2 min before eluting purified DNA in the elution buffer (150 *μ*L) for 6 min at 75°C. The V4 region of the 16S rRNA gene was PCR amplified using barcoded 515F/806R primers, followed by amplicon sequencing using the Illumina MiSeq v2 (San Diego, CA) to obtain 250-bp paired end reads, as described in detail previously [29]. Microbiome sequencing was performed at the National Cancer Institute Caner Genomics Research Laboratory (Bethesda, MD).

### Bioinformatic processing

Bioinformatic data processing was performed using the QIIME 2 pipeline [30]. Raw paired-end sequence reads were demultiplexed, filtered, merged, and processed into amplicon sequence variants (ASVs, i.e., 100% OTUs) using DADA2 (v1.6.0) [31]. Taxonomy was assigned to the ASVs using the SILVA v132 database [32]. A total of 2,067 unique ASVs remained after removing non-bacterial sequences. Alpha diversity metrics, including observed ASVs, Faith’s Phylogenetic Diversity (PD) [33], and the Shannon index [34], were calculated by taking the mean of 10 subsamples with rarefaction at 25,000 reads (S1 Fig in S1 File). Beta diversity matrices, including Bray-Curtis [35], unweighted UniFrac [36], and weighted UniFrac, were computed after rarefaction at 25,000 reads. Relative abundances were calculated from the phylum to genus levels without rarefaction. Taxa with prevalence less than 10% or mean relative abundance less than 0.002 were excluded, which resulted in 7 phyla, 11 classes, 16 orders, 22 families, and 30 genera for inclusion in subsequent statistical analyses. Five study samples were removed after rarefaction and one study sample was excluded due to low sample volume which left a total of 234 study samples for inclusion in the analysis.

For the chemostat community quality control samples, the coefficients of variation (CVs) were less than 8% for all alpha diversity metrics across the chemostat samples. The CVs for the relative abundances of the top four most abundant phyla were between 9–28% (Firmicutes 9.10%, Bacteroidetes 4.98%, Fusobacteria 14.3%, Proteobacteria 27.7%). Principal coordinates analysis (PCoA) plots generated from beta diversity matrices showed a clear separation between the quality control samples and the study samples (S2 Fig in S1 File). All blank samples were removed after rarefaction with an average of 79.4 reads (SD 95.7) per blank.

All sequencing data are available in the NCBI Sequence Read Archive under project accession number PRJNA909756, along with sample metadata including participant demographics and other covariates.

### Statistical analysis

Differences in the extracted DNA yields between ethanol-free and ethanol-containing mouthwashes and the different delayed processing times were assessed using the Wilcoxon signed-rank test and the Kruskal-Wallis test, respectively. The comparability of ethanol-free and ethanol-containing mouthwashes and the stability of the two mouthwash types after delayed processing were assessed based on alpha and beta diversity metrics and relative abundances of taxa. Alpha diversity metrics were compared between paired samples of ethanol-free mouthwash and ethanol-containing mouthwash using the Wilcoxon signed-rank test. Differences in alpha diversity metrics between delayed processing time points were assessed using linear mixed-effects models (lmer function in the lmerTest package); sample collection date (day 1 vs day 2) and processing delay time (0 vs 5 vs 10 days) were included as fixed effects and subjects were included as random effects, with each mouthwash modeled separately. PCoA was performed using beta diversity matrices to visually assess the separation of samples by mouthwash type and processing delay time. Differences in the microbial composition by the mouthwash type and processing delay time were tested using permutational multivariate analysis of variance (PERMANOVA, adonis2 function in the vegan R package) on the beta diversity matrices. Distance-based coefficients of determination (*R*^2^) were obtained using PERMANOVA to estimate the proportion of variability in the microbial composition explained by between-subject differences, sample collection date, mouthwash type, and sample processing delay time. The Wilcoxon signed-rank test with Bonferroni correction for multiple comparisons was used to test for differences in the untransformed relative abundances of taxa between paired samples of ethanol-free and ethanol-containing mouthwash from the phylum to genus level. To quantify the comparability of ethanol-free and ethanol-containing mouthwashes and the stability of each mouthwash during delayed processing, intraclass correlation coefficients (ICCs) were calculated for multiple microbiome metrics: alpha (i.e., observed ASVs, Faith’s PD, Shannon index) and beta diversity (i.e., principal coordinates [PCs] explaining >10% of the variation in beta diversity matrices), and relative abundances of the top four phyla (i.e., Firmicutes, Bacteroidetes, Proteobacteria, and Fusobacteria) and genera (i.e., *Streptococcus*, *Prevotella 7*, *Haemophilus*, and *Gemella*). Specifically, ICCs were estimated for each microbiome metric using linear mixed-effects models (lmer function in the lme4 R package): $ICC=\sigma_{b}^{2}/(\sigma_{b}^{2}+\sigma_{w}^{2})$, where $\sigma_{b}^{2}$ is the between-subject variance and $\sigma_{w}^{2}$ is the within-subject variance. To assess the comparability of ethanol-free and ethanol-containing mouthwashes, mouthwash type (ethanol-free vs ethanol-containing), sample collection date, and processing delay time were included as fixed effects and subjects were included as random effects. To assess the stability of ethanol-free and ethanol-containing mouthwashes during delayed processing, sample collection date and processing delay time were included as fixed effects and subjects were included as random effects, with each mouthwash modeled separately. All statistical analyses were performed using R Statistical Software (v4.1.2; R Core Team 2021), and statistical tests were performed at a significance level of 0.05.

## Results

### DNA yields

Overall, the extracted DNA yield was lower in ethanol-free mouthwash compared with ethanol-containing mouthwash. The median DNA yield was 1,343 (interquartile range [IQR] 1,791) ng for ethanol-free mouthwash and 1,803 (IQR 2,049) ng for ethanol-containing mouthwash (*P* < 0.0001, S3 Fig in S1 File). However, there was no significant difference in the amount of extracted DNA by the processing delay time. The median DNA yield was 1,517 (IQR 1,746) ng for immediately processed samples; 1,636 (IQR 1,949) ng for samples stored for 5 days; and 1,539 (IQR 1,779) ng for samples stored for 10 days (*P* = 0.830, S3 Fig in S1 File; samples from both mouthwash types combined for each time point); the DNA yield also did not differ by time point when stratifying by mouthwash type. There was a slight increase in the DNA yield on the second day of sample collection compared to the first day. On the first day of sample collection, the median DNA yield was 1,375 (IQR 1,979) ng, whereas on the second day the median DNA yield was 1,723 (IQR 1,713) ng (*P* = 0.0488, S4 Fig in S1 File; samples from both mouthwash types combined for each time point). However, this is unlikely to have biased the results for comparing the two mouthwashes since the order of the mouthwash type used for collection on each day of the two days was randomized.

### Comparability of ethanol-free and ethanol-containing mouthwashes

**Fig 1:**
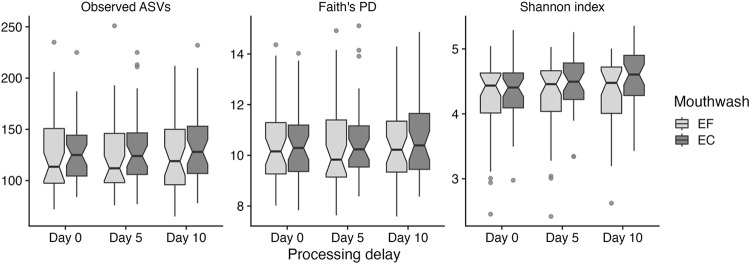
Comparison of alpha diversity metrics between ethanol-free (EF) and ethanol-containing (EC) mouthwashes by processing time. Medians are marked by the horizontal lines in the boxes. Observations outside 1.5 times the interquartile range above the upper quartile and below the lower quartile are shown as outliers. Notches represent 95% confidence intervals for comparing medians. Differences between the two mouthwash types were tested using the Wilcoxon signed-rank test. Significant differences between the two mouthwashes were detected for observed ASVs and the Shannon index for samples processed 5 days (observed ASVs: *P* = 0.0112; Shannon index: *P* = 0.000413) and 10 days (observed ASVs: *P* = 0.00721; Shannon index: *P* < 0.0001) after collection. No significant differences were observed for all other comparisons.

For samples processed and frozen immediately after collection, there were no differences in any of the alpha diversity metrics between ethanol-free and ethanol-containing mouthwash samples (*P* > 0.05, Table 1, Fig 1). For samples processed 5 days after collection, significant differences were observed between the two mouthwashes for observed ASVs (ethanol-free: median 116, IQR 46.0; ethanol-containing: median 124, IQR 40.5; *P* = 0.0112) and the Shannon index (ethanol-free: median 4.46, IQR 0.618; ethanol-containing: median 4.50, IQR 0.561; *P* = 0.000413), but not for Faith’s PD (ethanol-free: median 9.83, IQR 2.17; ethanol-containing: median 10.2, IQR 1.62; *P* = 0.114, Table 1, Fig 1). Similarly, for samples processed 10 days after collection, observed ASVs (ethanol-free: median 118, IQR 55.0; ethanol-containing: median 128, IQR 46.0; *P* = 0.00721) and the Shannon index (ethanol-free: median 4.46, IQR 0.693; ethanol-containing: median 4.61, IQR 0.617; *P* < 0.0001) were significantly different between the two mouthwashes, but there was no difference for Faith’s PD (ethanol-free: median 9.94, IQR 2.05; ethanol-containing: median 10.4, IQR 2.20; *P* = 0.149, Table 1, Fig 1).

**Table 1:** Comparison of alpha diversity metrics between ethanol-free (EF) and ethanol-containing (EC) mouthwashes by processing time.

	Processing delay (days)	EF, median (IQR)	EC, median (IQR)	*P*
Observed ASVs	0	113 (54.5)	124 (37.5)	0.771
	5	116 (46.0)	124 (40.5)	0.0112
	10	118 (55.0)	128 (46.0)	0.00721
Faith’s PD	0	10.1 (2.29)	10.3 (1.79)	0.742
	5	9.83 (2.17)	10.2 (1.62)	0.114
	10	9.94 (2.05)	10.4 (2.20)	0.149
Shannon index	0	4.43 (0.624)	4.39 (0.532)	0.215
	5	4.46 (0.618)	4.50 (0.561)	0.000413
	10	4.46 (0.693)	4.61 (0.617)	< 0.0001

*P*: obtained from Wilcoxon signed-rank tests

IQR: interquartile range

Ordination plots generated from PCoA performed using the Bray-Curtis and unweighted/weighted UniFrac beta diversity matrices did not show obvious visual separation between the ethanol-free and ethanol-containing mouthwashes (S5 Fig in S1 File). The first three PCs cumulatively explained 50.2% (PC1: 26.2%, PC2: 17.0%, PC3: 7.00%), 37.0% (PC1: 20.2%, PC2: 9.70%, PC3: 7.10%), and 80.0% (PC1: 58.0%, PC2: 14.9%, PC3: 7.10%) of the variation in the Bray-Curtis, unweighted UniFrac, and weighted UniFrac beta diversity matrices, respectively. Distance-based coefficient of determination (*R*^2^) calculated using permutational analysis of variance (PERMANOVA) showed that more than half of the variability in microbial composition was explained by between-subject differences (*R*^2^ > 0.50, *P* < 0.0001) and only a small proportion was explained by mouthwash type (*R*^2^ < 0.0015, *P* > 0.90, Table 2). The model residual showed that a large proportion of the variability (*R*^2^ > 0.40) was not explained by any of the variables included in the model (i.e., subject, mouthwash type, processing time, collection date; Table 2).

**Table 2:** Differences in the microbial composition by mouthwash type (ethanol-free vs.ethanol-containing) and processing delay time assessed using permutational multivariate analysis of variance (PERMANOVA).

	Bray-Curtis		Unweighted UniFrac		Weighted UniFrac	
	*R* ^*2*^	*P*	*R* ^*2*^	*P*	*R* ^*2*^	*P*
Collection date	0.00288	0.185	0.00254	0.401	0.00328	0.215
Mouthwash type	0.000983	0.947	0.00140	0.939	0.000519	0.933
Processing delay	0.00239	0.951	0.00194	1.00	0.00280	0.748
Subject	0.589	< 0.0001	0.521	< 0.0001	0.539	< 0.0001
Residual	0.404		0.472		0.454	

Proportion of microbial variability explained by the collection date, mouthwash type, processing time, and subject are represented by the distance-based coefficients of determination (*R*^*2*^).

**Fig 2:**
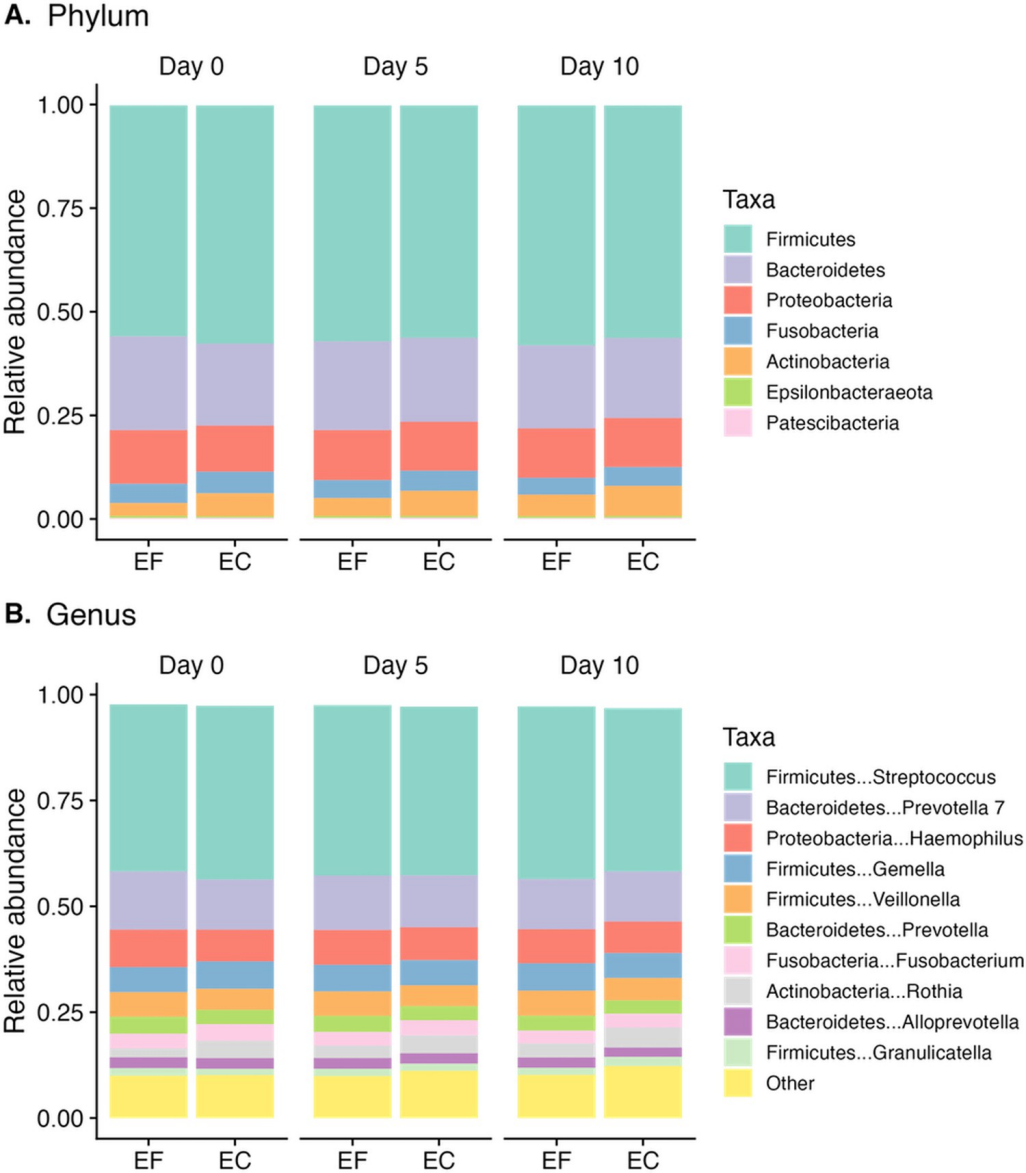
Taxonomic profiles at the phylum (A) and genus (B) levels comparing ethanol-free (EF) and ethanol-containing (EC) mouthwashes at each processing time point.

To visually compare the taxonomic profiles between the two mouthwashes, the mean relative abundance of each phylum (Fig 2A) and genus (Fig 2B) was calculated by the mouthwash type and processing time point. Overall, the taxonomic profiles of ethanol-free mouthwash and ethanol-containing mouthwash were similar to each other for all processing time points and at all taxonomic levels (Fig 2: phylum and genus levels, S6 Fig in S1 File: class to family level). However, at the individual-subject level, there were noticeable differences between the two mouthwashes for some subjects (S7 Fig in S1 File).

Relative abundances of many taxa were significantly different between ethanol-free and ethanol-containing mouthwashes at each taxonomic level (S8 Fig in S1 File, S1 Table). For example, while there were no differences in the relative abundances of the phyla Firmicutes, Proteobacteria, and Patescibacteria (all Bonferroni-corrected *P* [adj *P*] = 1.00), relative abundances of Bacteroidetes (mean difference 0.0155, SD 0.0623; adj *P* = 0.0144) and Epsilonbacteraeota (mean difference 0.000729, SD 0.00195; adj *P* = 0.00025) were higher and Fusobacteria (mean difference 0.00476, SD 0.0161; adj *P* = 0.000188) and Actinobacteria (mean difference 0.0200, SD 0.0312; adj *P <* 0.0001) were lower in ethanol-free mouthwash compared with ethanol-containing mouthwash. At the genus level, there were no differences in the relative abundances of the top five most abundant genera, which included *Streptococcus* (adj *P* = 1.00), *Prevotella 7* (adj *P* = 1.00), *Haemophilus* (adj *P* = 0.788), *Gemella* (adj *P* = 1.00), and *Veillonella* (adj *P* = 0.0833). Relative abundances of some of the less abundant genera were significantly different between the two mouthwashes, such as *Prevotella* (mean difference 0.00445, SD 0.0139; adj *P* = 0.0193), *Fusobacterium* (mean difference 0.00292, SD 0.0145; adj *P* = 0.0476), *Rothia* (mean difference 0.0153, SD 0.0291; adj *P* < 0.0001), *Leptotrichia* (mean difference 0.00186, SD 0.00619; adj *P* = 0.0118), and *Actinomyces* (mean difference 0.00273, SD 0.00679; adj *P* = 0.00261). For a full list of differences in relative abundances of taxa between the two mouthwashes at each taxonomic level, along with mean/median relative abundances in each mouthwash type, see S1 Table.

**Fig 3:**
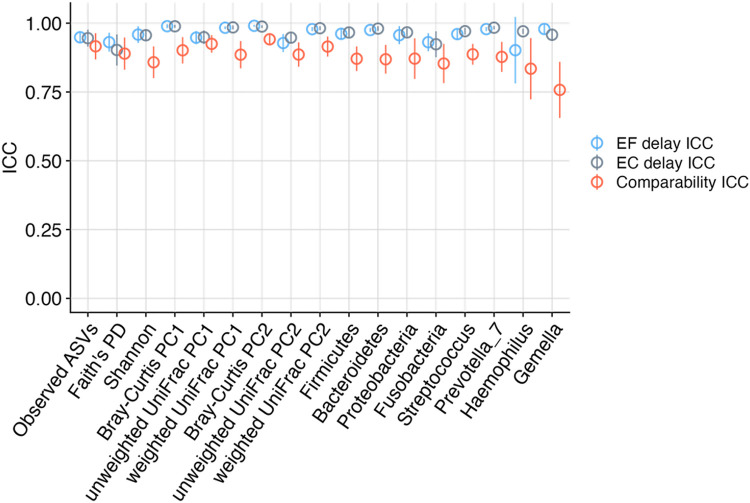
Intraclass correlation coefficients (ICCs) calculated for alpha and beta diversity metrics and the relative abundances of the top four abundant phyla and genera to assess the comparability of ethanol-free (EF) and ethanol-containing (EC) mouthwashes (comparability ICC) and the stability of each mouthwash type during delayed processing (delay ICC).

ICCs were calculated for various microbiome metrics to quantify the comparability of ethanol-free and ethanol-containing mouthwashes (Fig 3, S2 Table). The comparability of the two mouthwashes was generally high, with ICCs for alpha and beta diversity metrics greater than 0.85. ICCs for the relative abundances of the top four most abundant phyla (i.e., Firmicutes, Bacteroidetes, Proteobacteria, and Fusobacteria) and genera (i.e., *Streptococcus*, *Prevotella 7*, *Haemophilus*, and *Gemella*) were greater than 0.85 and 0.75, respectively.

### Sensitivity to delayed processing of ethanol-free and ethanol-containing mouthwashes

For ethanol-free mouthwash, no differences were observed over delayed processing time for alpha diversity measured by observed ASVs (*P* > 0.60) and Faith’s PD (*P* > 0.40, Table 1, S9 Fig in S1 File). There was also no difference in the Shannon index between immediately processed ethanol-free mouthwash samples and those processed 5 days after collection (*P* = 0.201). However, there was a significant increase in the Shannon index when comparing ethanol-free mouthwash samples processed immediately (median 4.43, IQR 0.624) and those processed 10 days after collection (median 4.46, IQR 0.693; *P* = 0.00299). For ethanol-containing mouthwash, all alpha diversity metrics showed an increase over delayed processing time. There was a marginal increase in observed ASVs between ethanol-containing mouthwash samples processed immediately upon collection (median 124, IQR 37.5) and those processed 5 days after collection (median 124, IQR 40.5; *P* = 0.0564), and observed ASVs further increased in samples processed 10 days after collection (median 128, IQR 46.0; *P* < 0.0001). For Faith’s PD, there was no difference between ethanol-containing mouthwash samples processed immediately and those processed 5 days after collection (*P* = 0.365). However, there was a significant difference in Faith’s PD between immediately processed ethanol-containing mouthwash samples (median 10.3, IQR 1.79) and those after 10 days of delayed processing (median 10.4, IQR 2.20; *P* = 0.0155). For the Shannon index, there was a significant and noticeable gradual increase at both 5 (median 4.50, IQR 0.561; *P* < 0.0001) and 10 days (median 4.61, IQR 0.617; *P* < 0.0001) of delayed processing compared with immediately processed (median 4.39, IQR 0.532) ethanol-containing mouthwash samples.

PCoA plots of beta diversity matrices did not show any differences in the microbial composition across the three processing time points (S10 Fig in S1 File). Based on the results from PERMANOVA, the proportion of variability in the microbial composition explained by the processing delay time was small, with *R*^2^ ~ 0.0020 (*P* > 0.70, Table 2).

Overall, taxonomic profiles were generally stable across the three processing time points for both mouthwashes (Fig 2 and S6 Fig in S1 File). Stability during delayed processing was also observed at the individual-subject level for both mouthwashes (S7 Fig in S1 File). The type of mouthwash appeared to have a greater influence on intra-individual variability of taxonomic profiles compared to the different processing time points.

Stability during delayed processing was high for both ethanol-free and ethanol-containing mouthwashes based on ICCs calculated for microbial metrics (Fig 3). ICCs of alpha and beta diversity metrics and relative abundances of the top four phyla and genera were all greater than 0.90 for both mouthwashes (Fig 3, S2 Table).

## Discussion

The primary aims of this study were to (1) compare oral washes collected with ethanol-free and ethanol-containing mouthwashes for microbiome analysis, and (2) evaluate the stability of the oral microbiome after storage for up to 10 days to assess the sensitivity of ethanol-free and ethanol-containing mouthwashes to delayed processing. For the first aim, the results showed that ethanol-free mouthwash generally performed very similarly to ethanol-containing mouthwash, with some differences in the relative abundances of certain taxa. However, no differences were observed in the overall microbial compositions of the two mouthwashes. For the second aim, both mouthwashes showed high stability during delayed processing based on alpha and beta diversity metrics, and relative abundances of the top four abundant phyla and genera. These results demonstrate that ethanol-free mouthwash can be used as an alternative to ethanol-containing mouthwash for collecting and mailing oral wash samples for oral microbiome studies.

While the microbiome profiles measured in ethanol-free and ethanol-containing mouthwashes were similar, there were some differences, particularly in the relative abundances of specific taxa, which may have been due to differences in the chemical ingredients of the mouthwashes. The specific brands of ethanol-free and ethanol-containing mouthwashes used in this study both contained the ingredient, cetylpyridinium chloride, a quaternary ammonium compound frequently used as an antimicrobial agent in over-the-counter oral care products to reduce plaque accumulation and gingival inflammation [37, 38]. The antimicrobial activity of cetylpyridinium chloride is likely to have inhibited bacterial growth in the oral wash samples, which may have led to the high stability during delayed processing for the two mouthwashes in the present study. It is possible that the presence of ethanol in the ethanol-containing mouthwash and the lack thereof in the ethanol-free mouthwash may have contributed to the observed differences between the two mouthwashes. Ethanol is typically added in mouthwashes as a solvent for active ingredients and flavors and also as a preservative for its antiseptic properties [24]. Ethanol tolerance varies considerably by bacteria [39, 40], but the presence of ethanol (15 wt% in Scope®) may have impacted the relative abundance of certain bacteria in the ethanol-containing mouthwash. Various antimicrobial agents are used in other commercially available mouthwashes (e.g., chlorhexidine, essential oils, hydrogen peroxide, etc.) [24], for both ethanol-containing and ethanol-free options, and it is possible that each mouthwash may perform somewhat differently for oral microbiome analyses.

For both ethanol-free and ethanol-containing mouthwashes, more than half of the variability in microbial composition was due to inter-individual differences, but a large proportion (> 40%) of the remaining was not explained by any of the other experimental factors considered in this study, which included mouthwash type, processing time, and collection date (Table 2). The composition of the oral microbiome has been shown to be influenced by various factors, such as age [41], host genetics [42–44], and sociodemographic and lifestyle factors including diet [45], geography [46], cohabitation [47], and race/ethnicity [46, 48]. However, these factors may only account for a relatively small proportion of the inter-individual variability of the oral microbiome [49], and a more important contributor may be oral health [45]. Microbes found in the supra- and subgivingival communities are directly involved in the development of dental caries and periodontal disease [5], the two most common oral diseases, and the overall composition of the oral microbiome may differ substantially based on the oral health status of an individual. We did not account for these various individual factors that may have contributed to the unexplained variability in the microbiome composition observed in our study, but this did not affect the primary goal of this study, which was to compare microbiome measurements from ethanol-containing and ethanol-free mouthwash since both mouthwash types were collected from the same individual.

The type of mouthwash used for oral wash collections should be consistent for all subjects and time points within a given epidemiologic study whenever possible as using a mix of different mouthwashes may introduce systematic errors. For example, in our study, there was a significant difference in observed ASVs between ethanol-free and ethanol-containing mouthwashes when sample processing was delayed by 5 days, with a difference of 5 ASVs (ethanol-free: 125 vs ethanol-containing: 130); and this difference in mean observed ASVs increased to 10 after 10 days of delayed processing (ethanol-free: 125 vs ethanol-containing: 135). In a recent case-cohort study nested within three, prospective cohort studies in the United States that evaluated associations between the oral microbiome and the risk of developing lung cancer, mean observed ASVs differed by approximately 10 between lung cancer cases and the referent subcohort within each of the three cohorts at baseline, and this was found to be a statistically significant difference in that analysis [50]. Since the magnitude of the differences in mean observed ASVs due to mouthwash type and those due to biologically meaningful differences appear to be similar, this indicates that using a mix of mouthwash types for oral wash collections may lead to biased results or mask true differences between groups in epidemiologic studies of the oral microbiome. To minimize these potential issues, at least one consistent mouthwash, whether it be ethanol-free or ethanol-containing, should be selected for future oral wash collections. If that were not possible, alternative analytic methods which account for these systematic differences would need to be considered to make valid inferences from such data.

In many epidemiologic studies, particularly large-scale studies where participants are recruited from a large geographical area, self-administered collection procedures provide a logistically and economically feasible means to obtain samples from study participants. The mouthwash collection method is simple, inexpensive, noninvasive, and can be performed at home by the participants themselves without supervision by trained personnel. In an ideal setting, samples are chilled, transported to the laboratory for processing, and frozen until analysis immediately after collection. However, this is not possible when sample collection is performed at home or in other settings that are not within close proximity to laboratories. Realistically, sample processing is often delayed for multiple days from the time of collection to when the samples are received in the laboratory. Our results demonstrate that ethanol-free mouthwash can accommodate delayed processing, as seen by the stability of the oral microbiome in ethanol-free mouthwash samples for up to 10 days in conditions mimicking cold shipment warming up to ambient temperatures over time. Compared with ethanol-containing mouthwash, it is possible that ethanol-free mouthwash may provide more stability during delayed processing for some microbiome metrics, such as for alpha diversity, as seen in the present study. Furthermore, ethanol-free mouthwash samples can be shipped safely by mail and stored without concerns about flammability since they do not contain ethanol. Therefore, for future epidemiologic studies, investigators should consider oral wash collections using ethanol-free mouthwash, which has the same advantages of being low cost and easy to collect but without some of the challenges of ethanol-containing mouthwash.

Our study has several strengths and limitations. We directly compared Crest® Scope® mouthwash, the most widely used mouthwash for epidemiologic investigations, with an ethanol-free alternative from the same brand (Crest® Pro-Health™) using multiple microbial metrics commonly measured in microbiome studies. The two mouthwashes were compared within the same individual, and the order of collection was randomized across participants to prevent any bias due to one mouthwash type consistently being collected before the other. We also assessed the stability of both mouthwashes during delayed processing to account for potential shipping delays that may occur during mail-in collections. Our results are likely to be generalizable to other populations–an exception may be unhealthy individuals since our study population only included healthy individuals. One limitation is that we did not compare multiple types of ethanol-containing and ethanol-free mouthwashes that included different active ingredients. Another potential limitation of our study is that we did not assess the stability of the oral microbiome in the mouthwashes at temperatures higher than ambient temperature. Therefore, it is unclear how processing delays will impact mouthwash samples shipped in hot climates. Furthermore, we only evaluated bacterial communities using 16S rRNA sequencing, so it is unclear how the mouthwashes compare using other sequencing methods (e.g., shotgun metagenomic sequencing) or amplicon sequencing of other kingdoms (e.g., internal transcribed spacer gene sequencing for fungi). We also did not assess the long-term stability after freezing (i.e., months, years) for measuring the oral microbiome in mouthwashes, which may have important implications for establishing biorepositories of oral wash samples.

## Conclusion

In this study, we compared the use of ethanol-free mouthwash and ethanol-containing mouthwash for oral microbiome studies. We found that the two mouthwashes performed similarly overall, but there were differences in the relative abundances of some phyla and genera. We also found that both mouthwashes demonstrated high stability during delayed processing of up to 10 days without freezing, before the samples were processed in the laboratory. These results indicate that ethanol-free mouthwash is suitable as an alternative to ethanol-containing mouthwash for collecting oral wash samples and shipping by mail in epidemiologic studies.

## Supporting information

S1 Table Comparison of relative abundances of taxa between ethanol-free (EF) and ethanol-containing (EC) mouthwash from the phylum to genus level. Differential abundance was tested using the Wilcoxon signed-rank test with Bonferroni correction for multiple testing.
(XLSX)

S2 Table Intraclass correlation coefficients estimated for alpha and beta diversity metrics and relative abundances of the top four abundant phyla and genera to quantify the comparability of ethanol-free (EF) and ethanol-containing (EC) mouthwashes and to assess the processing delay stability of each mouthwash type. (XLSX)

S1 File Supplemental figures (S1–10 Figs). (DOCX)

S2 File STORMS checklist. (XLSX)

S3 File Data dictionary for data submitted to the NCBI Sequence Read Archive. (XLSX)
