# Supplementary material for: Evaluation of alcohol-free mouthwash for studies of the oral microbiome
Source: PLoS One. 2023 Apr 27;18(4):e0284956. doi: 10.1371/journal.pone.0284956 (PMC10138257; doi:10.1371/journal.pone.0284956)

**Supporting information**

**Evaluation of alcohol-free mouthwash for studies of the oral microbiome**

Yukiko Yano^1^, Emily Vogtmann^1^, Alaina H. Shreves^1,2^, Stephanie J. Weinstein^1^, Amanda Black^1^, Norma Diaz-Mayoral^3^, Yunhu Wan^1^, Weiyin Zhou^1,3^, Xing Hua^4^, Casey Dagnall^1,3^, Amy Hutchinson^1,3^, Kristine Jones^1,3^, Belynda D. Hicks^1,3^, Kathleen Wyatt^1^, Nicolas Wentzensen^1^, Christian C. Abnet^1^

1. Division of Cancer Epidemiology & Genetics, National Cancer Institute, Bethesda, Maryland
2. Department of Epidemiology, Harvard T.H. Chan School of Public Health, Boston, Massachusetts
3. Leidos Biomedical Research, Inc., Frederick National Laboratory for Cancer Research, Frederick, Maryland
4. Public Health Sciences Division, Fred Hutchinson Cancer Research Center, Seattle Washington

**S1 Fig**. Rarefaction curve showing the mean number of observed species by sample type: ethanol-free (EF) and ethanol-containing (EC) mouthwash study samples, and quality control samples (chemostat and sequencing control). Error bars represent 95% confidence intervals.


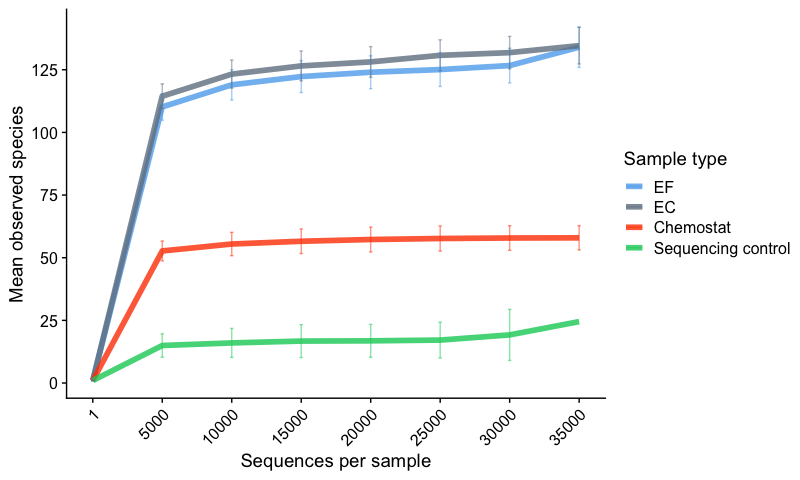


**S2 Fig.** Plots of the first three principal coordinates from principal coordinate analysis using the Bray-Curtis (top), unweighted UniFrac (middle), and weighted UniFrac (bottom) beta diversity matrices including study samples and quality control samples (i.e., chemostat community and sequencing control).


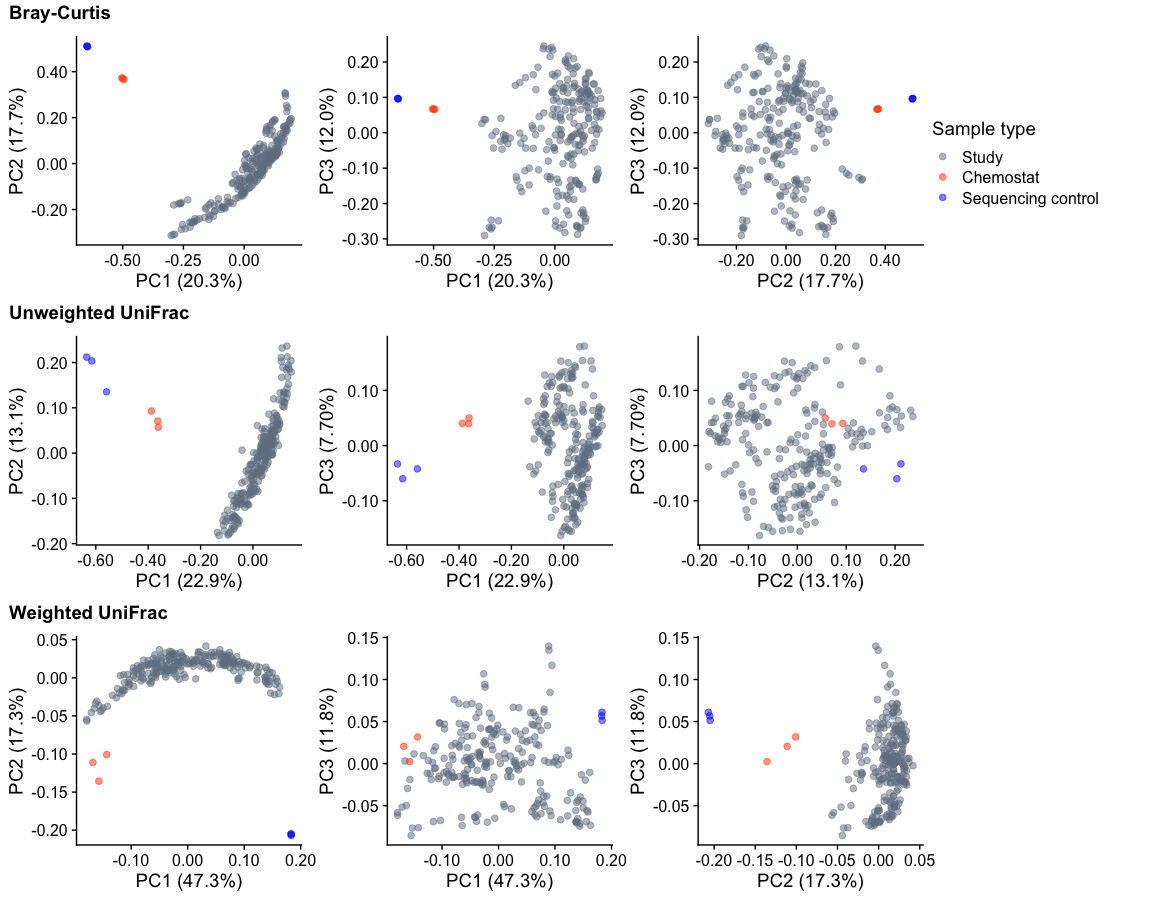


**S3 Fig.** Amount of extracted DNA by processing delay time for ethanol-free (EF) and ethanol-containing (EC) mouthwash.


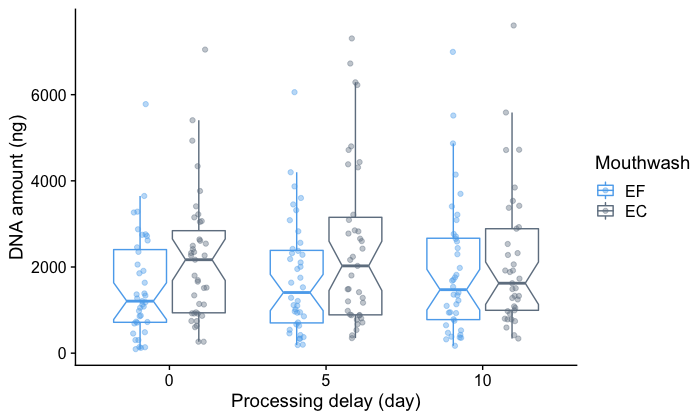


**S4 Fig.** Amount of extracted DNA by collection date for ethanol-free (EF) and ethanol-containing (EC) mouthwash.


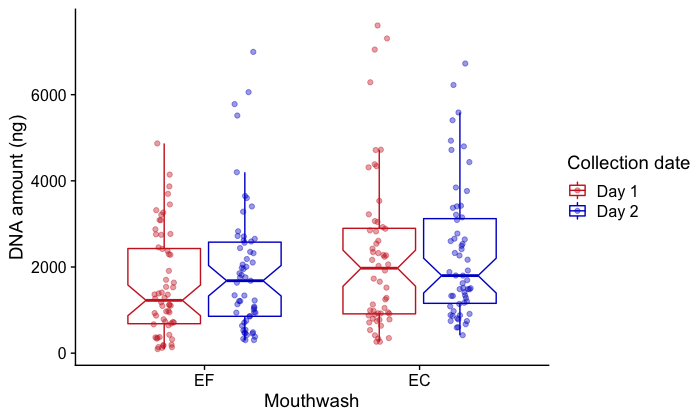


**S5 Fig.** Plots of the first three principal coordinates from principal coordinate analysis using the Bray-Curtis (top), unweighted UniFrac (middle), and weighted UniFrac (bottom) beta diversity matrices comparing ethanol-free (EF) mouthwash and ethanol-containing (EC) mouthwash.


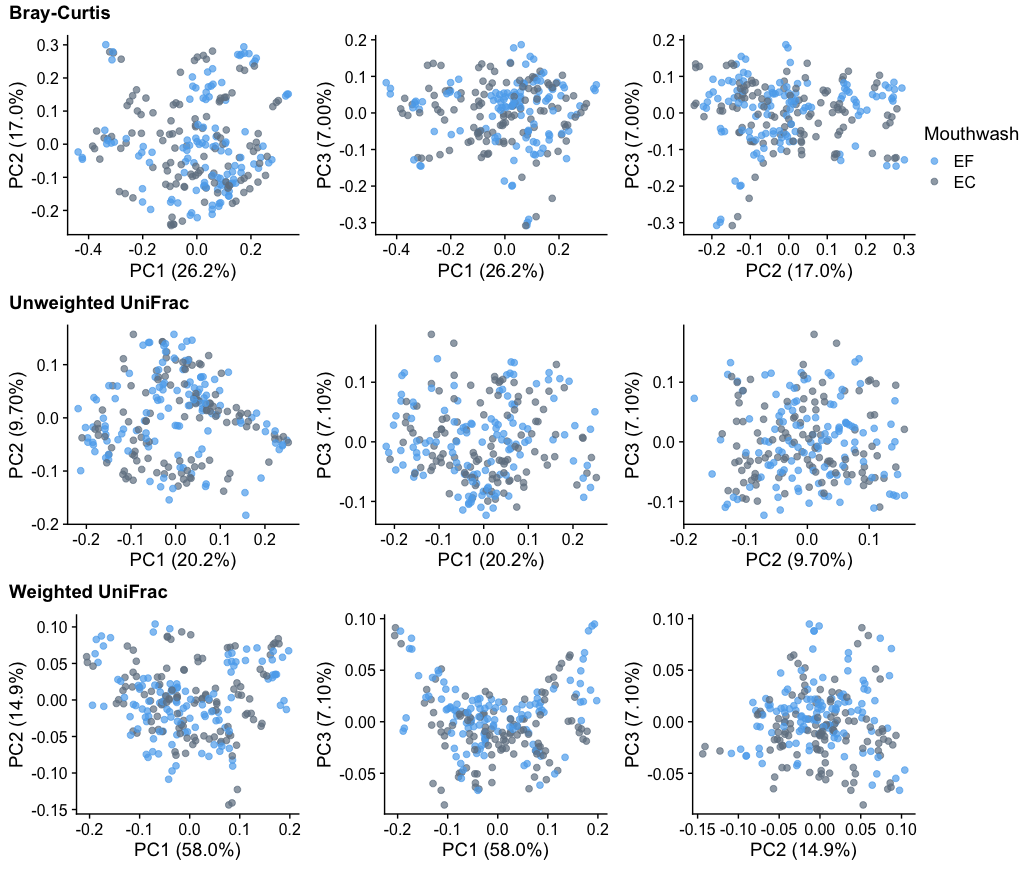


**S6 Fig.** Taxonomic profiles at the class (A), order (B), and family (C) levels comparing ethanol-free (EF) and ethanol-containing (EC) mouthwashes at each processing time point.


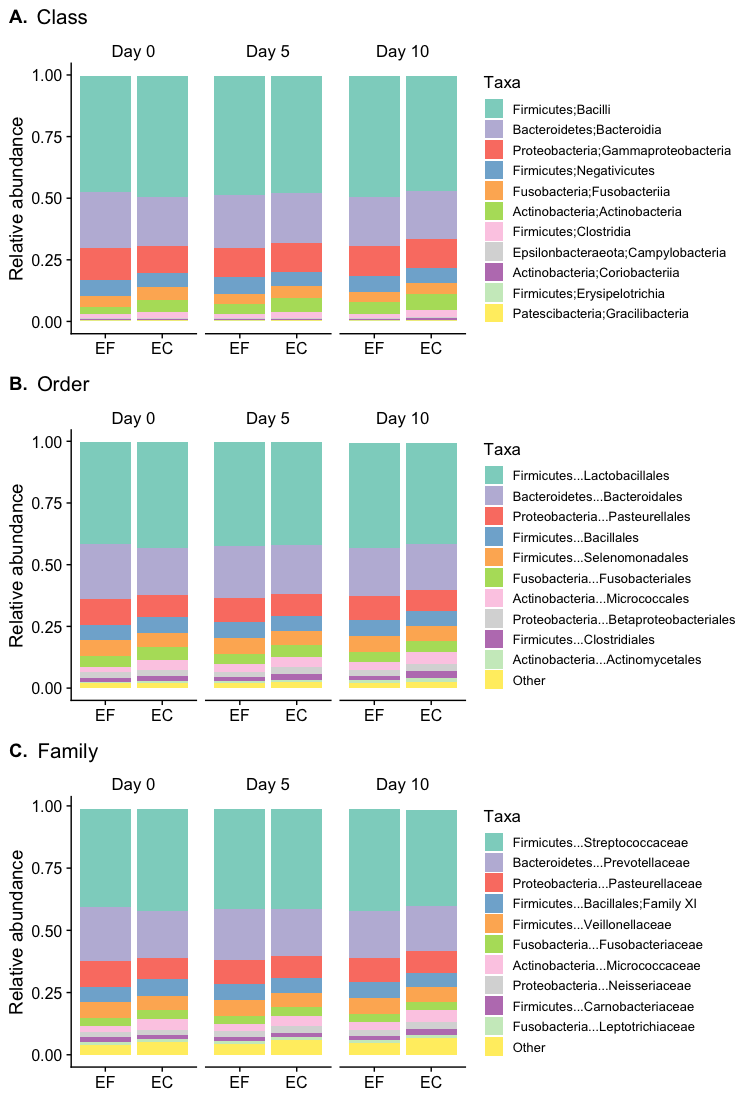


**S7 Fig.** Taxonomic profiles at the phylum (A) and genus (B) levels comparing ethanol-free (EF) and ethanol-containing (EC) mouthwashes at each processing time point for each subject.


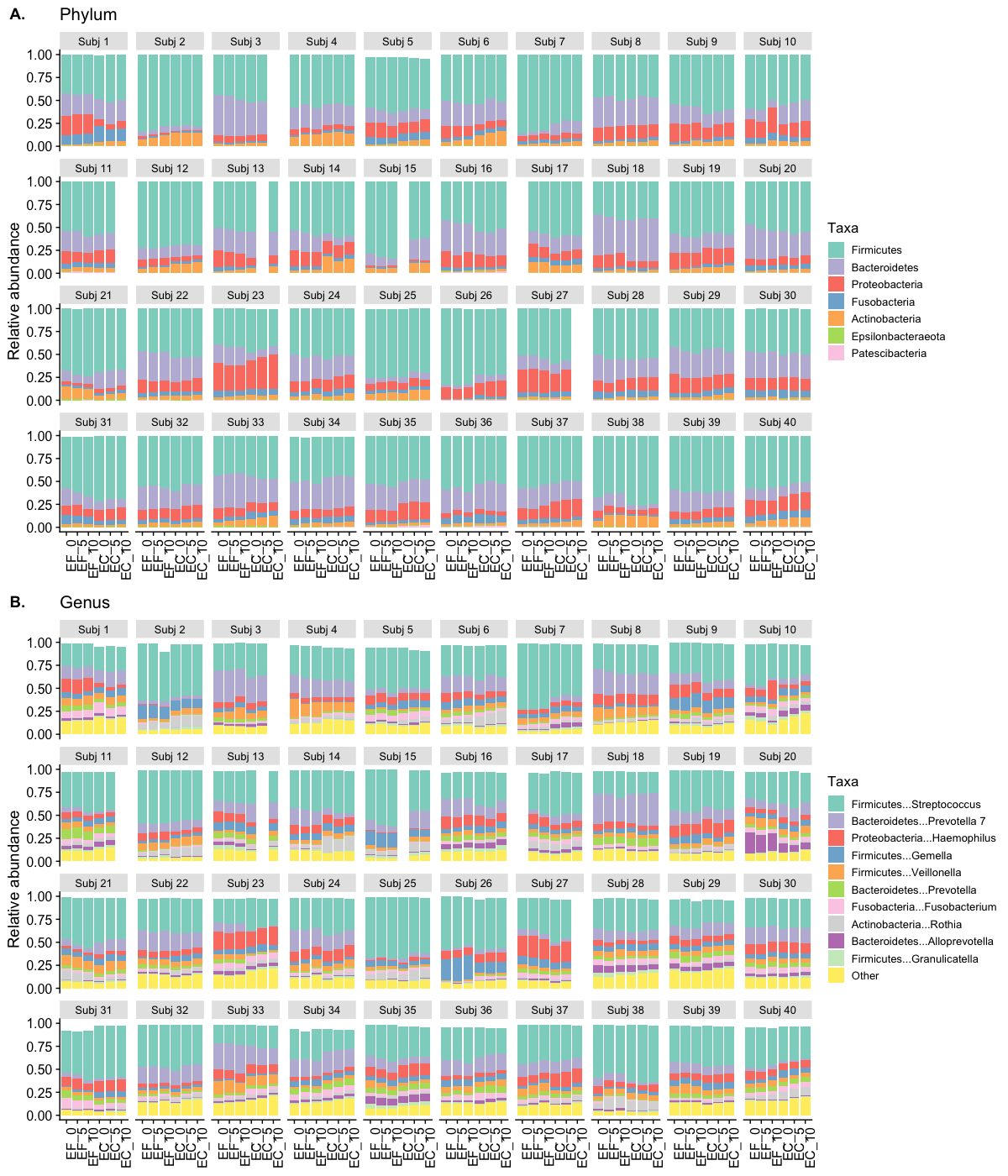


**S8 Fig.** Differences in relative abundances of taxa from the phylum to genus levels between ethanol-containing (EC) and ethanol-free (EF) mouthwashes. Difference of 0 indicates no difference in relative abundances between EF and EC mouthwashes. Boxplots in red indicate significant differences in relative abundances between EF and EC mouthwashes based on Wilcoxon signed-rank tests with Bonferroni correction for multiple testing.


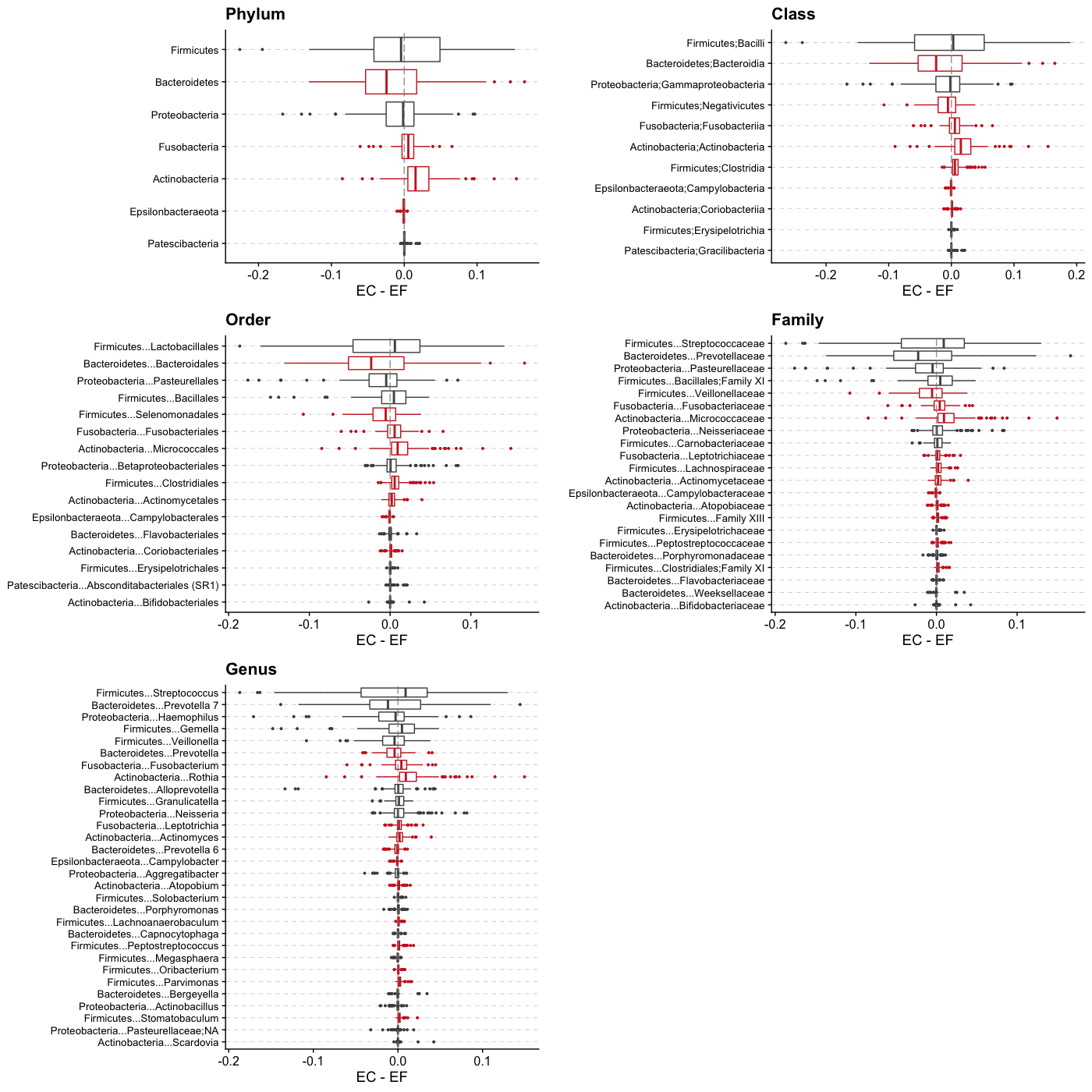


**S9 Fig.** Comparison of alpha diversity metrics between the three processing time points (0, 5, and 10 days after collection) by mouthwash type (ethanol-free [EF], ethanol-containing [EC]). Medians are marked by the horizontal lines in the boxes. Observations outside 1.5 times the interquartile range above the upper quartile and below the lower quartile are shown as outliers. Notches represent 95% confidence intervals for comparing medians. Differences between the processing time points were tested using linear mixed-effects models, where samples immediately processed upon collection were used as the reference to compare samples processed 5 and 10 days after collection. For EF mouthwash, no differences were observed for observed ASVs and Faith’s PD at all processing time points. Although there was no difference in the Shannon index in EF mouthwash samples after 5 days of delayed processing, there was a significant increase on day 10 (*P* = 0.00299). For EC mouthwash, all alpha diversity metrics showed a significant increase after 10 days of delayed processing compared with immediately processed samples (*P* < 0.05). After 5 days of delayed processing, there was a slight increase in observed ASVs (*P* = 0.0564) and a larger increase in the Shannon index (*P* < 0.0001), whereas Faith’s PD remained stable in EC mouthwash samples.


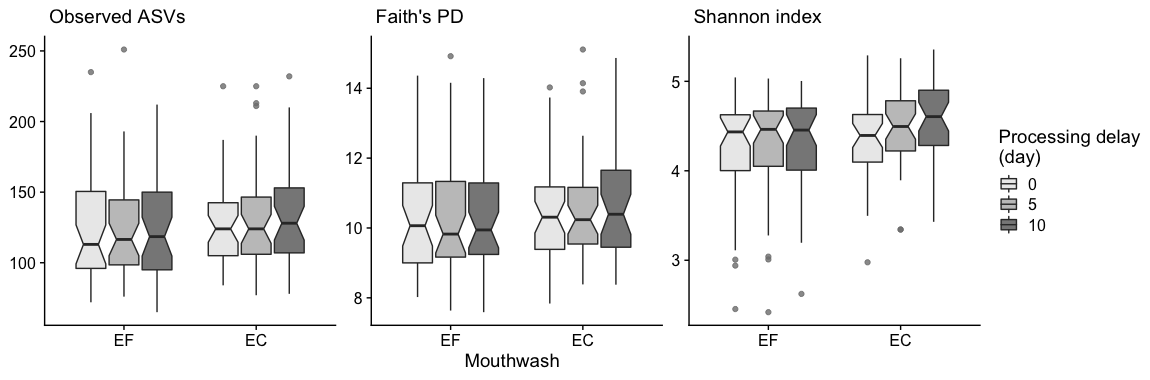


**S10 Fig.** Plots of the first three principal coordinates from principal coordinate analysis using the Bray-Curtis (top), unweighted UniFrac (middle), and weighted UniFrac (bottom) beta diversity matrices comparing the three processing times (0, 5, and 10 days after collection).


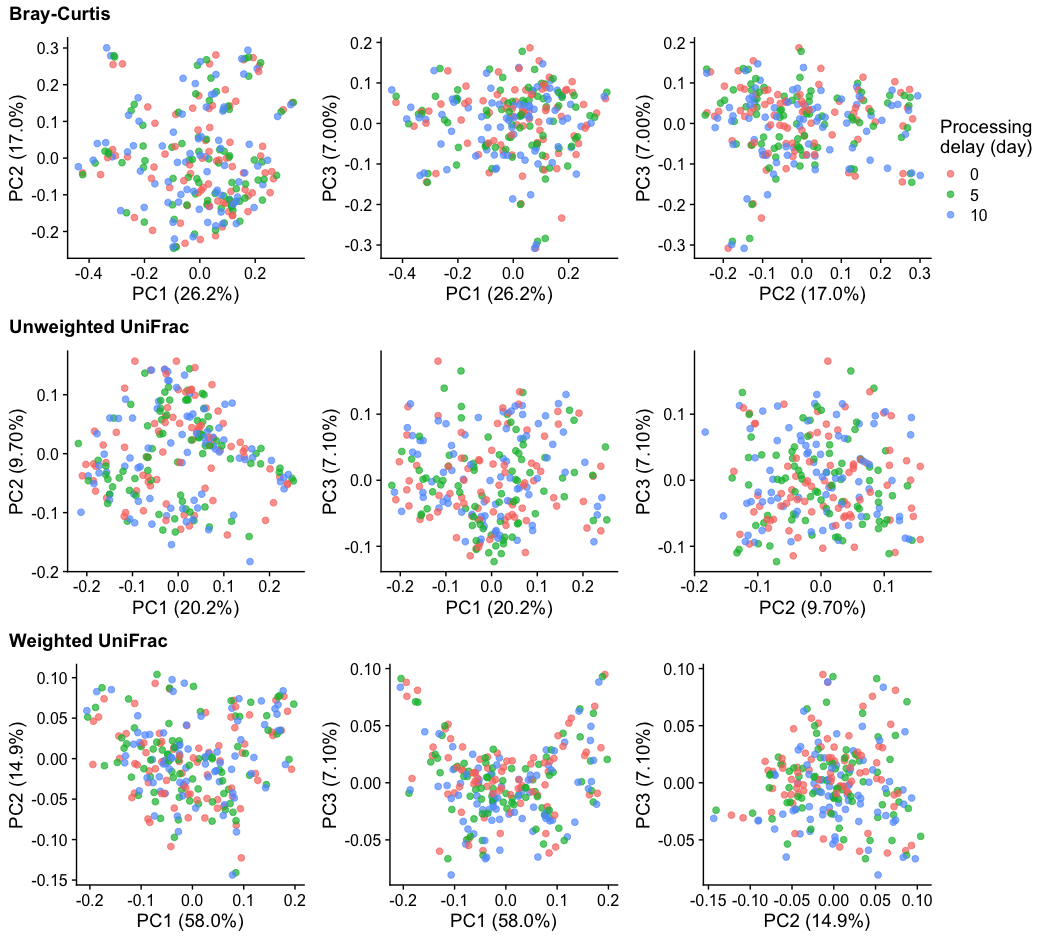

Supplement: S1 File — (DOCX) [file pone.0284956.s003.docx]
